# Computational Design of FastFES Treatment to Improve Propulsive Force Symmetry During Post-stroke Gait: A Feasibility Study

**DOI:** 10.3389/fnbot.2019.00080

**Published:** 2019-10-01

**Authors:** Nathan R. Sauder, Andrew J. Meyer, Jessica L. Allen, Lena H. Ting, Trisha M. Kesar, Benjamin J. Fregly

**Affiliations:** ^1^Computational Biomechanics Laboratory, Department of Mechanical and Aerospace Engineering, University of Florida, Gainesville, FL, United States; ^2^Neuromechanics Laboratory, Wallace H. Coulter Department of Biomedical Engineering, Emory University and Georgia Institute of Technology, Atlanta, GA, United States; ^3^Motion Analysis Laboratory, Department of Rehabilitation Medicine, Emory University School of Medicine, Atlanta, GA, United States; ^4^Rice Computational Neuromechanics Laboratory, Department of Mechanical Engineering, Rice University, Houston, TX, United States

**Keywords:** fast treadmill training, functional electrical stimulation, neuromusculoskeletal modeling, computational modeling, direct collocation optimal control, paretic propulsion, stroke, muscle synergies

## Abstract

Stroke is a leading cause of long-term disability worldwide and often impairs walking ability. To improve recovery of walking function post-stroke, researchers have investigated the use of treatments such as fast functional electrical stimulation (FastFES). During FastFES treatments, individuals post-stroke walk on a treadmill at their fastest comfortable speed while electrical stimulation is delivered to two muscles of the paretic ankle, ideally to improve paretic leg propulsion and toe clearance. However, muscle selection and stimulation timing are currently standardized based on clinical intuition and a one-size-fits-all approach, which may explain in part why some patients respond to FastFES training while others do not. This study explores how personalized neuromusculoskeletal models could potentially be used to enable individual-specific selection of target muscles and stimulation timing to address unique functional limitations of individual patients post-stroke. Treadmill gait data, including EMG, surface marker positions, and ground reactions, were collected from an individual post-stroke who was a non-responder to FastFES treatment. The patient's gait data were used to personalize key aspects of a full-body neuromusculoskeletal walking model, including lower-body joint functional axes, lower-body muscle force generating properties, deformable foot-ground contact properties, and paretic and non-paretic leg neural control properties. The personalized model was utilized within a direct collocation optimal control framework to reproduce the patient's unstimulated treadmill gait data (verification problem) and to generate three stimulated walking predictions that sought to minimize inter-limb propulsive force asymmetry (prediction problems). The three predictions used: (1) Standard muscle selection (gastrocnemius and tibialis anterior) with standard stimulation timing, (2) Standard muscle selection with optimized stimulation timing, and (3) Optimized muscle selection (soleus and semimembranosus) with optimized stimulation timing. Relative to unstimulated walking, the optimal control problems predicted a 41% reduction in propulsive force asymmetry for scenario (1), a 45% reduction for scenario (2), and a 64% reduction for scenario (3), suggesting that non-standard muscle selection may be superior for this patient. Despite these predicted improvements, kinematic symmetry was not noticeably improved for any of the walking predictions. These results suggest that personalized neuromusculoskeletal models may be able to predict personalized FastFES training prescriptions that could improve propulsive force symmetry, though inclusion of kinematic requirements would be necessary to improve kinematic symmetry as well.

## Introduction

Approximately 15 million people experience a stroke each year (MacKay and Mensah, 2004), with walking dysfunction being one of the most common sequelae (Lloyd-Jones et al., 2010; Verma et al., 2012). Stroke-related walking disability has been associated with a host of co-morbidities including hypertension, heart disease, diabetes, and cognitive decline (Ostwald et al., 2006; Abellan van Kan et al., 2009; Mutikainen et al., 2011; Ostir et al., 2013; Garcia-Pinillos et al., 2016; Rosso et al., 2017; Savica et al., 2017) resulting in a decreased quality of life and increased risk of death (Nor Azlin et al., 2016). While stroke rehabilitation treatments often restore some level of walking function (Balaban et al., 2011), they rarely restore walking ability to a pre-stroke level (Bogey and Hornby, 2007). Stroke-induced walking deficits primarily affect one side of the body, resulting in a slow and asymmetric gait pattern (Verma et al., 2012) characterized by neural control changes (Lamontagne et al., 2007) and compensatory motion patterns. For example, post-stroke gait is often characterized by reduced paretic leg propulsion during stance phase (McGinley et al., 2006) and decreased paretic leg toe clearance from the ground during swing phase (Verma et al., 2012). These changes lead to inefficient compensatory strategies such as increased propulsive force generation on the non-paretic side and hip hiking to facilitate toe clearance on the paretic side.

Fast-speed treadmill training with functional electrical stimulation (FastFES) is a promising treatment for improving walking ability post-stroke. However, little data currently exist for determining the best way to customize the treatment to individual-specific gait deficits. The rationale for FastFES training is that fast speed walking by itself can improve gait biomechanics in individuals post-stroke, while task-specific electrical stimulation of selected muscles can provide feedback to the nervous system to promote motor learning of the appropriate timing and activation of the stimulated muscles (Kesar et al., 2011; Awad et al., 2014, 2016). The FastFES intervention has been shown to improve gait function and energy cost of gait in individuals with chronic post-stroke hemiparesis (Awad et al., 2014, 2016). This combination may help the damaged central nervous system adapt favorably to the new post-stroke reality. However, selection of muscles to stimulate, along with stimulation timing and amplitude, are currently standardized based on the normal activation profile of muscles during gait, clinical intuition, and the subject's tolerance to electrical stimulation. Furthermore, during FastFES, only two muscles are typically targeted for stimulation—tibialis anterior and gastrocnemius—due to technical limitations. Tibialis anterior is stimulated to improve paretic toe clearance during swing phase, and gastrocnemius is stimulated to improve paretic propulsion at the end of stance phase (Allen et al., 2018). Timing patterns of the stimulation are usually constrained to simple on/off cycles based on foot switch signals.

Though stroke affects each patient's neural control capabilities differently, current standardized FastFES treatment does not account for this reality. Patient specific coordination deficits suggest the need for patient-specific treatment prescriptions (Allen et al., 2018). Inter-individual variability in post-stroke sensorimotor impairments may explain why some patients respond to standardized treatment while others do not. Non-responders could potentially experience greater treatment efficacy if different muscles were stimulated, or if the standard muscles were stimulated with different timing. However, no method currently exists for predicting *a priori* which two muscles are the best targets for stimulation, and how they should be stimulated, to achieve the maximal improvement in walking function for any particular patient.

An emerging approach for addressing the treatment personalization problem is personalized neuromusculoskeletal modeling. If key parameter values in a neuromusculoskeletal model are personalized to the unique anatomical, physiological, and neurological characteristics of a specific patient, then the resulting personalized model could potentially be used to predict and even optimize an individual patient's functional outcome for different treatment scenarios under consideration (Fregly et al., 2012; Meyer et al., 2016). Several modeling studies have already analyzed or optimized various aspects of muscle electrical stimulation, including electrode shape in epiretinal stimulation (Cao et al., 2015), stimulation site selection for hand opening (De Marchis et al., 2016), stimulation pulse duration and polarity for de-enervated muscles (Pieber et al., 2015), stimulation profiles and lower limb trajectories to improve FES- and orthosis-based walking (Sharma et al., 2014), and stimulation timing for foot drop correction (Azevedo Coste et al., 2014). However, no study to date has used a personalized neuromusculoskeletal model to design a personalized FastFES treatment tailored to the functional limitations of a specific individual post-stroke.

This study evaluates the feasibility of designing a personalized FastFES treatment protocol using a personalized neuromusculoskeletal model coupled with direct collocation optimal control. The subject studied was a non-responder to the standard FastFES treatment protocol (Allen et al., 2018), making him an excellent candidate for computational exploration of alternative muscle stimulation protocols that theoretically could improve his treatment outcome. The treatment design problem was framed as a direct collocation optimal control problem that minimized propulsive force asymmetry between the two legs while making minimal changes to the subject's non-stimulated neural control strategy, which was modeled using subject-specific muscle synergies. Propulsive force symmetry was targeted for improvement since recent studies have shown that it is an important determinant of walking ability (Bowden et al., 2006; Schmid et al., 2007). The computational treatment design process involved personalizing key parameters in a full-body neuromusculoskeletal walking model to treadmill walking data collected from the subject, and then using the personalized model to solve a sequence of direct collocation optimal control problems. An initial optimal control problem verified that the personalized model could be used to predict the subject's unstimulated muscle activations, joint kinematics, and ground reactions. The remaining optimal control problems predicted how the subject would walk when two paretic leg muscles were stimulated in three ways: (1) Standard muscle selection with standard stimulation timing, (2) Standard muscle selection with optimized stimulation timing, and (3) Optimized muscle selection with optimized stimulation timing. The results demonstrate the feasibility of using this computational treatment design approach for identifying new avenues of clinical exploration.

## Materials and Methods

### Experimental Data Collection

We collected treadmill gait data from an individual post-stroke (age >70 years, ~8 years after stroke) who was a non-responder to the standard FastFES treatment protocol. The subject gave written informed consent, and the study was approved by the institutional review boards of Emory University and the University of Florida. Collected data included full-body video motion capture data (Vicon, Centennial, CO, USA), bilateral force plate data from a split-belt instrumented treadmill with belts tied (Bertec Corporation, Columbus, OH, USA), and surface EMG data from 14 muscles per leg (Table 1; 28 total signals) (Konigsberg Instruments, Pasadena, CA, USA). The subject had a slow self-selected walking speed of 0.3 m/s and a visually asymmetric gait pattern, with the paretic right leg exhibiting stereotypical hip hiking. The same subject was participant NR1 in a recently published FastFES clinical study (Allen et al., 2018), although the data used in the present study (which were much more extensive) were collected more than a year after completion of the clinical study. Notes taken during the clinical study indicated that further investigation into the causes of non-response and corresponding alterations to treatment design were needed for this subject.

**Table 1:** List of muscles present in each leg of the neuromusculoskeletal model, including muscles with measured EMG signals (Measured), muscles whose EMG signals were copied from neighboring muscles with similar anatomical function (Copied), and muscles whose EMG signals were predicted using synergy signals extracted from measured EMG signals (Predicted).

**Muscle**	**Abbreviation**	**Measured**	**Copied**	**Predicted**
Adductor brevis	AddBrev		X	
Adductor longus	AddLong		X	
Adductor magnus (Distal)	AddMagDist		X	
Adductor magnus (Ischial)	AddMagIsch		X	
Adductor magnus (Mid)	AddMagMid	X		
Adductor magnus (Proximal)	AddMagProx		X	
Gluteus maximus 1	GlutMax1	X		
Gluteus maximus 2	GlutMax2		X	
Gluteus maximus 3	GlutMax3		X	
Gluteus medius 1	GlutMed1	X		
Gluteus medius 2	GlutMed2		X	
Gluteus medius 3	GlutMed3		X	
Gluteus minimus 1	GlutMin1		X	
Gluteus minimus 2	GlutMin2		X	
Gluteus minimus 3	GlutMin3		X	
Tensor fasciae latae	TFL	X		
Semimembranosus	Semimem		X	
Semitendinosus	Semiten	X		
Biceps femoris long head	BifemLH	X		
Biceps femoris short head	BifemSH		X	
Rectus femoris	RecFem	X		
Vastus medialis	VasMed	X		
Vastus lateralis	VasLat	X		
Vastus intermedius	VasInt		X	
Gastrocnemius lateralis	GasLat	X		
Gastrocnemius medialis	GasMed	X		
Tibialis anterior	TibAnt	X		
Peroneus brevis	PerBrev		X	
Peroneus longus	PerLong	X		
Peroneus tertius	PerTert		X	
Soleus	Sol	X		
Iliopsoas	IP			X
Tibialis posterior	TibPost			X
Extensor digitorum longus	EDL			X
Flexor digitorum longus	FDL			X

Experimental data were collected for several types of trials. Static trial data were collected in which the subject stood upright with an anatomically neutral joint alignment for several seconds with feet pointing forward. Data from this static trial were used for scaling an initial generic musculoskeletal model and determining the locations of reflective surface markers on the model (see below). Six different isolated joint motion trials were performed to facilitate calibration of lower-body joint positions and orientations in the body segments. One isolated joint motion trial was performed for each hip, knee, and ankle, where each trial exercised all functional axes for the selected joint (Reinbolt et al., 2005, 2008). Gait trials were performed at the subject's fastest comfortable walking speed of 0.6 m/s without FES, which was the speed used for the subject's previous FastFES training. To help maintain balance during the treadmill gait trials, the subject rested his hands on a handlebar suspended from the ceiling. One representative gait trial was selected for subsequent computational modeling and optimization efforts. The selection process involved identifying gait cycles with clean surface marker, ground reaction, and EMG data, eliminating cycles near the start and end of the trial where transient conditions were present, and finally determining the one gait cycle whose period was closest to the mean.

### Computational Model Personalization

We personalized a generic full-body musculoskeletal model (Hamner et al., 2010) developed in OpenSim (Delp et al., 2007; Seth et al., 2018) to the unique anatomical, physiological, and neurological characteristics of the subject using the subject's experimental movement data. The generic model possessed 44 lower-body muscles, of which 36 were retained, and 37 degrees-of-freedom (DOFs), including three DOF hip joints, one DOF knee joints, and two DOF ankle joints. As a preliminary task, the generic OpenSim model was scaled to the subject's dimensions using surface marker data from the static trial and OpenSim's Scale Model tool. Three mutually perpendicular forces and moments were added to each hand in the model to account for the subject's hands resting on a handlebar. In addition, a backpack was added to the torso of the model to account for EMG system hardware. Muscles controlled the hips, knees, and ankles of the model, while net torque actuators controlled the lower back joint and the two shoulder, elbow, and toes joints. Activation dynamics, Hill-type muscle models with rigid tendons (De Groote et al., 2016), surrogate musculoskeletal geometry models, and deformable foot-ground contact models were implemented in Matlab (the Mathworks, Natick, MA, USA) for use within the OpenSim skeletal model.

As described briefly below, model personalization involved a four-step calibration process performed in Matlab with calls to OpenSim analyses through Matlab Mex functions and OpenSim's C++ API. Traditional optimization problems used to calibrate model parameter values were solved with either the “lsqnonlin” or “fmincon” optimizer in Matlab, while direct collocation optimal control problems used to calibrate model parameter values and controls and to predict new gait motions were solved with GPOPS-II optimal control software for Matlab (Patterson and Rao, 2014) using the IPOPT optimizer (Wächter and Biegler, 2006). The four steps in the model personalization process were similar to the ones presented in a recent study (Meyer et al., 2016), which provides further details on the process.

#### Joint Model Personalization

The first step involved personalization of the model's lower-body functional axes using data from the isolated joint motion trials and selected gait trial combined with repeated OpenSim “Inverse Kinematics” analyses. Only pelvis and lower body marker motion data were needed for this step.

To perform this model personalization step, we formulated an optimization problem that sought to calibrate parameters defining the positions and orientations of the lower body joints (hips, knees, and ankles) in their respective body segments as well as parameters defining the positions and orientations of marker triads placed on the pelvis, thighs, shanks, and feet of the model. The cost function minimized the sum of squares of errors between experimental and model-predicted marker positions using all motion trials together. Each function evaluation performed an OpenSim “Inverse Kinematics” analysis to calculate the current marker location errors. Matlab's “lsqnonlin” non-linear least squares algorithm was used to perform the optimization. The calibrated joint and marker triad positions and orientations were applied to the model and used in a final OpenSim “Inverse Kinematics” analysis to determine joint position, velocity, and acceleration time histories for subsequent steps of the model personalization process.

#### Muscle-Tendon Model Personalization

The second step involved personalization of the model's EMG-driven muscle-tendon force and moment generating properties using data from 40 gait cycles and OpenSim “Inverse Dynamics” and “Muscle” analyses. The data needed for this step included joint kinematics found as in the first step along with ground reaction and EMG data. Three tasks were performed in preparation for this model personalization step. First, EMG data from 40 gait cycles were processed (high-pass filtered, demeaned, rectified, and low-pass filtered) as described in Meyer et al. (2016), resulting in envelopes of muscle excitation. Second, an OpenSim “Inverse Dynamics” analysis was performed to calculate the net hip, knee, and ankle joint moments to be matched by muscle forces estimated by this step of the model personalization process. Third, an OpenSim “Muscle” analysis was performed repeatedly to calculate muscle-tendon lengths and moment arms for each muscle over a wide range of sampled combinations of lower-body joint positions. The sampled quantities were fitted simultaneously as polynomial functions of joint positions as described in Meyer et al. (2016), thereby producing surrogate representations of the subject's musculoskeletal geometry for rapid calculation of muscle-tendon lengths, velocities, and moment arms.

Once these preparatory tasks were completed, we formulated an optimization problem that sought to calibrate Hill-type muscle-tendon model parameters (scale factors for EMG normalization, electromechanical delays, activation dynamics time constants, activation non-linearization shape factors, optimal muscle fiber lengths, and tendon slack lengths) along with parameters defining the surrogate musculoskeletal geometry. The cost function minimized the weighted sum of squares of errors between three types of lower-body quantities: (1) inverse dynamic and model-predicted total joint moments, (2) published experimentally measured (Silder et al., 2007) and model-predicted passive joint moments, and (3) initial and current model parameter values as regularization terms (i.e., terms that minimized changes in parameter values away from their initial guesses to make the optimal solution unique). Joint moment errors were calculated for hip flexion-extension and adduction-abduction, knee flexion-extension, and ankle plantarflexion-dorsiflexion and inversion-eversion. Twice as much weight was placed on hip moment errors as on knee and ankle moment errors to produce comparable magnitude errors at all three joints. No OpenSim analyses were needed for function evaluations. Matlab's “fmincon” sequential quadratic programming algorithm was used to perform the optimization. The calibrated muscle activations were required for the final step of the model personalization process.

Because fine-wire EMG data were not available for important deep muscles (i.e., iliopsoas, tibialis posterior, extensor digitorum longus, and flexor digitorum longus), we incorporated muscle synergy techniques into the optimization process to estimate the activations for the 4 muscles in each leg with missing EMG signals (Bianco et al., 2017). Synergy analysis of the subject's muscle activations revealed that only 2 or 3 synergies (depending on normalization method) were needed to achieve 95% total VAF for each leg. However, since our muscle synergies were not simply fitting EMG data but rather making the subject's personalized model walk in a dynamically consistent manner that closely matched all available experimental data, we wanted all muscle activations in the model to be reconstructed with at least 95% VAF. Consequently, we chose to control each leg with 5 synergies, which was the number required to surpass the 95% individual muscle VAF threshold and ensure enough flexibility for constructing the shapes of the missing muscle activations.

**Figure 1:**
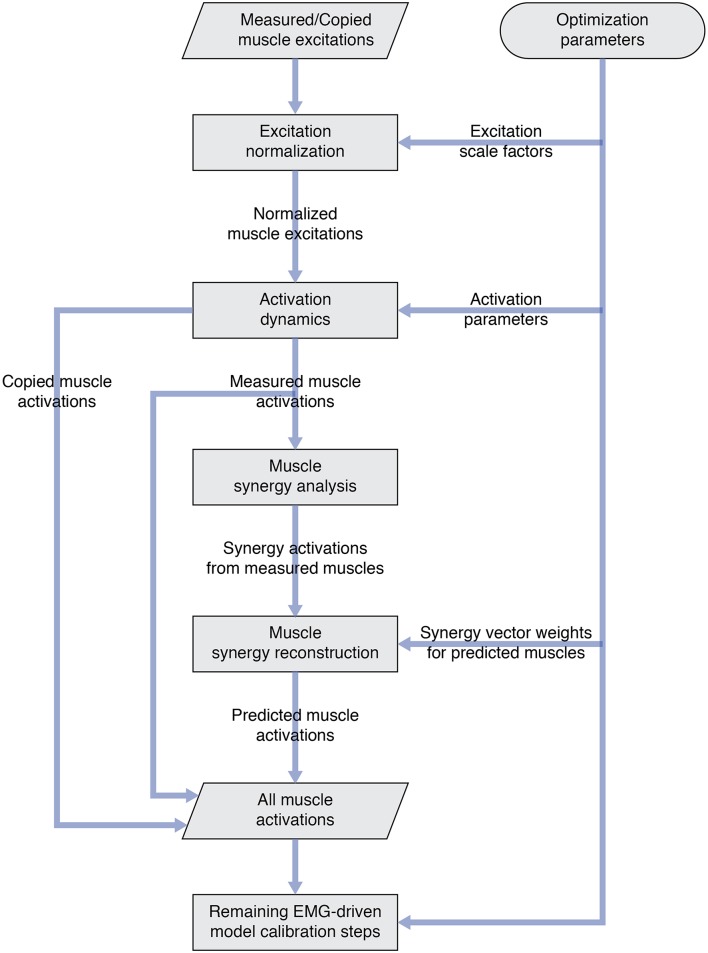
Flowchart showing modifications to the original EMG-driven model personalization process to accommodate muscles with missing EMG signals. Two new steps—“Muscle synergy analysis” and “Muscle synergy reconstruction”—were added to the existing process to predict missing muscle activations whose shapes were consistent with synergy activations extracted from muscles with measured EMG signals.

Once the number of synergies was selected, we used muscle synergy concepts to extend our EMG-driven model personalization process (Meyer et al., 2017) to the case where 4 important muscles per leg had missing EMG signals. The original personalization process was developed using a full set of EMG signals, where every muscle in the lower body model had either a measured EMG signal or an EMG signal that could be copied from a neighboring muscle with similar anatomical function (e.g., the semimembranosus EMG signal was copied from the semitendinosus EMG signal). Thus, no EMG signals needed to be predicted. To accommodate missing EMG signals, we added two new steps to the personalization process immediately after activation dynamics (Figure 1). The first new step performed muscle synergy analysis via non-negative matrix factorization (Lee and Seung, 1999; Tresch et al., 1999; Ting and Chvatal, 2010) on the 14 muscle activations per leg with associated measured EMG signals. This step produced 5 time-varying synergy activations that were assumed to apply to the 4 muscles with missing EMG signals (Bianco et al., 2017). The second new step performed muscle synergy reconstruction by multiplying the 5 time-varying synergy activations extracted in the previous step with the optimization's current guess for the 5 synergies ×4 unknown synergy vector weights/synergy = 20 synergy vector weights per leg for muscles with missing EMG signals. These synergy vector weights were new parameters added to the optimization problem formulation. The second step yielded 4 predicted muscle activations consistent with the optimization's current guess for excitation scale factors, activation parameters, and synergy vector weights for predicted muscles. The 4 muscle activations predicted for each leg were used in the current optimization iteration and updated in future iterations based on the latest values of the optimization parameters.

#### Ground Contact Model Personalization

The third step involved personalization of the model's foot-ground contact properties using data from the selected gait trial combined with repeated OpenSim “Point Kinematics” and “Inverse Dynamics” analyses. The data needed for this step included inverse dynamic joint moments found in the second step along with marker motion and ground reaction data. Compressive non-linear spring-dampers were distributed over a rectangular grid on the bottom of each two-segment foot model (Neptune et al., 2000). Overall grid dimensions were defined using a foot outline generated with a marker pointer (Jackson et al., 2016). Contact elements whose center points were inside the foot outline were retained, while those whose center points were outside of the foot outline were discarded. Contact elements were split between the rear foot and toes segments according to the segment in which they resided. The normal force generated by each contact element was a function of the element's penetration and penetration rate into the floor, while the frictional force generated by each contact element was a function of the element's normal force and slip velocity relative to the floor (Meyer et al., 2016).

To perform this model personalization step, we formulated a direct collocation optimal control problem that sought to calibrate parameters defining the stiffness, damping, and frictional properties of the non-linear spring-dampers on the bottom of each foot (Table 2, Model Personalization Problem 1.1; Meyer et al., 2016). The cost function minimized the weighted sum of squares of errors between four types of quantities: (1) experimental and model-predicted marker positions, (2) experimental and model-predicted ground reaction forces and moments, (3) lower-body inverse dynamic and model-predicted joint moments (no muscles were used in this step), and (4) inverse kinematic and model-predicted toe joint angles. The last term was included since a small error in toe marker position could produce a large change in ground reactions, making the foot-ground contact model calibration process much more difficult. Three mutually perpendicular forces and moments were applied to each hand to approximate the loads applied to the hands by the handlebar. Path constraints were used to ensure that the full-body skeletal dynamic equations were satisfied to within the specified tolerance of 1e-6. Each function evaluation performed OpenSim “Point Kinematics” and “Inverse Dynamics” analyses to calculate the current errors in ground reactions, marker positions, and joint moments (cost function) and in skeletal dynamics (constraints). Since skeletal dynamics were evaluated in an inverse rather than forward sense, we added joint jerk controls to the problem formulation to provide explicit forward dynamic equations (e.g., joint jerk is the first time derivative of joint acceleration, etc.) as required by GPOPS-II. Thus, the state vector consisted of joint positions, joint velocities, and joint accelerations.

**Table 2:** Overview of direct collocation optimal control problem formulations for the neuromusculoskeletal model personalization and FastFES treatment optimization process.

	**Cost function**	**Constraints**	**Controls**	**Static parameters**
**1 MODEL PERSONALIZATION**				
1.1 Calibrate foot-ground contact model to reproduce experimental data	Track experimental marker, ground reaction, joint moment, and toe angle data	Satisfy skeletal dynamics	Joint jerk; hand loads	Foot-ground contact model parameters
1.2 Generate dynamically consistent motion using calibrated foot-ground contact model	Track experimental marker, ground reaction, and joint moment data; minimize joint jerk	Satisfy skeletal dynamics; bound toe angle error; enforce ground reaction and joint angle periodicity	Joint jerk; hand loads	None
1.3 Calibrate synergy vectors and activations to reproduce experimental motion, ground reaction, and EMG data	Track experimental joint angle, ground reactions, joint moment, and muscle activation data; minimize joint jerk and hand loads	Satisfy skeletal dynamics; match OpenSim lower body joint moments using synergy controls; bound joint angle, ground reaction, and hand position errors; enforce periodicity and unit magnitude synergy vectors	Joint jerk; hand loads; synergy activations	Synergy vector weights
1.4 Verify calibrated model reproduces experimental motion and ground reactions without tracking any experimental quantities	Minimize joint jerk	Satisfy skeletal dynamics; match OpenSim lower body joint moments using synergy controls; bound hand position and synergy activation errors; enforce periodicity	Joint jerk; synergy activations	None
**2 TREATMENT OPTIMIZATION**				
2.1 *Baseline*—Add AP force asymmetry minimization to verification cost function	Minimize joint jerk and AP force asymmetry	Satisfy skeletal dynamics; match OpenSim lower body joint moments using synergy controls; bound hand position and synergy activation errors; enforce periodicity	Joint jerk; synergy activations	None
2.2 *Standard muscles/standard timing*—Add TibAnt and GasMed stimulation with experimental timing	Minimize joint jerk and AP force asymmetry	Satisfy skeletal dynamics; match OpenSim lower body joint moments using synergy controls; bound hand position, synergy activation, and stimulation timing errors; enforce periodicity	Joint jerk; synergy activations	Stimulation amplitude and timing
2.3 *Standard muscles/optimal timing*—Use TibAnt and GasMed stimulation with free timing	Minimize joint jerk and AP force asymmetry	Satisfy skeletal dynamics; match OpenSim lower body joint moments using synergy controls; bound hand position and synergy activation errors; enforce periodicity	Joint jerk; synergy activations	Stimulation amplitude and timing
2.4 Find optimal combination of two stimulated muscles	Minimize joint jerk and AP force asymmetry	Satisfy skeletal dynamics; match OpenSim lower body joint moments using synergy controls; bound hand position and synergy activation errors; enforce periodicity; limit number of stimulated muscles to two	Joint jerk; synergy activations	Stimulation amplitude and timing for all paretic leg muscles
2.5 *Optimal muscles/optimal timing*—Find optimal stimulation of two identified muscles	Minimize joint jerk and AP force asymmetry	Satisfy skeletal dynamics; match OpenSim lower body joint moments using synergy controls; bound hand position and synergy activation errors; enforce periodicity	Joint jerk; synergy activations	Stimulation amplitude and timing

Model personalization required solving four separate optimal control problems, while treatment optimization involved solving five separate optimal control problems, each of which used a full-body walking model developed in OpenSim and Matlab.

Using the model with calibrated foot-ground contact parameter values, we solved a subsequent direct collocation optimal control problem to generate a dynamically consistent walking motion that closely tracked experimental marker, ground reaction, and inverse dynamic joint moment data (Table 2, Model Personalization Problem 1.2). The problem formulation was identical to the previous one except that foot-ground contact model parameter values were fixed to their calibrated values, minimization of joint jerk controls and the three mutually perpendicular forces and moments applied to each hand were added to the cost function, and bounds were placed on allowable toe angle errors and on ground reaction and joint angle periodicity errors. The results of this problem were used as the starting point for the final model personalization step.

#### Neural Control Model Personalization

The final step involved personalization of the model's neural control properties using data from the selected gait trial combined with repeated OpenSim “Point Kinematics” and “Inverse Dynamics” analyses. The data needed for this step were the same as for the previous step except for the addition of muscle activations produced by the second step.

To perform this final model personalization step, we formulated a direct collocation optimal control problem that sought to calibrate parameters defining synergy vector weights and controls defining synergy activations (Table 2, Model Personalization Problem 1.3). Similar to Meyer et al. (2016), five muscle synergies were used to construct 36 muscle activations per leg. The cost function minimized the sum of squares of errors between four types of quantities: (1) inverse kinematic and model-predicted joint positions, (2) experimental and model-predicted ground reaction forces and moments, (3) lower-body inverse dynamic and model-predicted joint moments, and (4) EMG-driven and synergy-constructed muscle activations. The cost function again included regularization terms that minimized joint jerk controls and the three mutually perpendicular forces and torques applied to each hand. Path constraints ensured that the full-body skeletal dynamic equations were satisfied, lower body joint moments calculated from inverse skeletal dynamics matched corresponding joint moments calculated from synergy activations, and the hands remained on the handlebars in their experimentally measured positions. Each function evaluation performed OpenSim “Point Kinematics” and “Inverse Dynamics” analyses to calculate the current errors in ground reactions and joint moments (cost function) and in skeletal dynamics and hand positions (constraints). This final calibration optimization yielded muscle synergy controls that closely reproduced not only the subject's muscle activations but also his experimental marker motion and ground reaction data while also producing a dynamically consistent full-body walking motion. The results of this optimization served as the starting point for all subsequent optimal control problems that explored different FastFES treatment scenarios.

#### Complete Model Verification

To gain confidence in the complete personalized model, we solved a verification optimal control problem to demonstrate that we could predict the subject's unstimulated walking motion, joint moments, ground reactions, and muscle activations without tracking any of these quantities in the cost function or bounding any of these quantities in the path constraints (Table 2, Model Personalization Problem 1.4). The problem formulation used path constraints to bound synergy activation changes and hand position errors and terminal constraints to enforce motion and ground reaction periodicity. Changes in synergy activations were limited by path constraints rather than tracking terms in the cost function to guarantee a solution with only small changes in synergy activations. No changes in calibrated synergy vector weights were permitted, and applied hand forces and torques were defined to match those found by neural control model personalization. Thus, the only term in the cost function was minimization of joint jerk controls. The verification problem predicted a walking motion that essentially represented the results of a forward dynamic simulation using the complete personalized model.

### FastFES Treatment Optimizations

We used the subject's personalized neuromusculoskeletal model and direct collocation optimal control to predict the theoretically achievable improvement in anterior-posterior (AP) force symmetry for three FastFES treatment scenarios: (1) Standard muscle selection with standard stimulation timing, (2) Standard muscle selection with optimized stimulation timing, and (3) Optimized muscle selection with optimized stimulation timing. These three treatment scenarios built upon a baseline treatment optimization that predicted the theoretically achievable improvement in AP force symmetry under unstimulated conditions so that the effects of electrical stimulation could be isolated.

Each FastFES treatment optimization built upon a baseline treatment optimization with no electrical stimulation (see below) by adding simulated electrical stimulation to two selected paretic leg muscles. For each treatment optimization, simulated electrical stimulation was added on top of the subject's simulated unstimulated muscle activations. Electrical stimulation waveforms were assumed to be simple step functions defined by an on-time *t*_*on*_, off-time *t*_*off*_, and amplitude *A*. Since our optimal control prediction problems used controls related to muscle activation (i.e., the output of activation dynamics) rather than muscle excitation (i.e., the input to activation dynamics), we developed a closed-form equation that approximated the amplitude and shape of the activation output produced by activation dynamics (He et al., 1991) when given a step function excitation input:

(1) $$\begin{array}{l} a_{stim}(t)=\frac{A}{2}\{\tanh\left(c_{1}\left(t-t_{on}-t_{offset1}\right)\right)\\ \;-\tanh\left(c_{2}\left(t-t_{off}-t_{offset2}\right)\right)\}\\ \;+\frac{A}{2}\left\{1-\tanh\left(c_{2}\left(t-t_{off}-t_{offset2}+t_{end}\right)\right)\right\} \end{array}$$

In this equation, *a*_*stim*_(*t*) is the amplitude of activation produced by electrical stimulation at the current time *t*, *t*_*end*_ is the final time of the gait cycle, and *c*_1_, *c*_2_, *t*_*offset*1_, and *t*_*offset*2_ are adjustable parameters. For any stimulated muscle, we used non-linear least squares optimization to calibrate the four parameters *c*_1_, *c*_2_, *t*_*offset*1_, and *t*_*offset*2_ to match the output of the muscle's activation dynamics as closely as possible given a step function input of amplitude one. Thus, given *A*, *t*_*on*_, and *t*_*off*_ for any muscle, Equation (1) with calibrated parameters was used to define the muscle's time-varying activation from electrical stimulation. The form of Equation (1) also allows for electrical stimulation to extend beyond the end of the gait cycle and wrap around into the start of the same gait cycle. The total activation of a stimulated muscle was assumed to be the sum of its activation from electrical stimulation and its activation from muscle synergies, where the sum was constrained to be less than one. Furthermore, the maximum activation from electrical stimulation was bounded to be ≤ 0.7 so that total activation would never exceed one.

#### Baseline Treatment Optimization With No Stimulation

As a starting point for treatment optimization, we formulated and solved a baseline optimal control problem to quantify AP force asymmetry in the absence of electrical stimulation but with minimization of AP force asymmetry added to the cost function (Table 2, Treatment Optimization Problem 2.1). The optimal control problem formulation was identical to that of the verification problem except for the addition of a cost function term that minimized the squared difference in AP force impulse between the two legs. The weight on the AP force asymmetry term was chosen to be as large as possible without visibly affecting the predicted motion. This problem formulation allowed AP force asymmetry to be reduced primarily through changes in initial conditions, which were the initial positions and velocities of the skeletal model generalized coordinates, rather than through changes in muscle activations. The AP force asymmetry produced by this baseline problem served as the reference for quantifying improvements produced by the three FastFES treatment scenarios.

#### FastFES Treatment Optimization With Standard Muscles and Standard Timing

The first treatment scenario used standard paretic leg muscle selection—tibialis anterior (TibAnt) and medial gastrocnemius (GasMed)—with standard stimulation timing (Table 2, Treatment Optimization Problem 2.2). This optimal control problem assessed how stimulation amplitude for the standard muscles could affect propulsive force asymmetry. The main optimization parameters were stimulation amplitude for both muscles. Stimulation on-time *t*_*on*_ for both muscles was constrained to be within ±0.05 s of the standard experimental on-time, stimulation duration was fixed to the experimental duration, and stimulation off-time *t*_*off*_ was set to on-time plus duration.

#### FastFES Treatment Optimization With Standard Muscles and Optimized Timing

The second FastFES treatment scenario used standard muscle selection with optimized stimulation timing (Table 2, Treatment Optimization Problem 2.3). This optimal control problem assessed how altered stimulation timing for the standard muscles could affect propulsive force asymmetry. The problem formulation was the same as for the first FastFES treatment scenario except that bounds on stimulation on-time were eliminated so that stimulation amplitude, on-time, and duration for both muscles became the main optimization parameters.

#### FastFES Treatment Optimization With Optimized Muscles and Optimized Timing

The third FastFES treatment scenario used optimized muscle selection with optimized stimulation timing. To predict the outcome of this treatment scenario, we followed a two-step process: First, we predicted which two muscles to stimulate, and second, we predicted when and how much they should be stimulated. For the first step, we solved an optimal control problem that identified which two paretic leg muscles should be stimulated to achieve the maximum reduction in AP force asymmetry (Table 2, Treatment Optimization Problem 2.4). Muscles without EMG data were not candidates for stimulation, since their unstimulated activations were not known with certainty. Muscles that shared EMG data between multiple heads used shared stimulation properties, leaving 25 muscles for the selection process. The stimulation properties of these 25 muscles were defined by 75 adjustable parameters that accounted for stimulation amplitude *A*, on-time *t*_*on*_, and duration, which fixed stimulation off-time *t*_*off*_. A terminal constraint was added to force the optimization to select only two muscles. Since gradient-based optimizations require continuous functions, a continuous approximation to the number of stimulated muscles *n* was constructed as a function of the stimulation amplitude *A*_*i*_ of each muscle:

(2) $$\begin{array}{r} n=\overset{25}{\underset{i=1}{\sum }}\left(1-e^{-4Ai}\right) \end{array}$$

This approximation was constrained to be less than or equal to two plus a small tolerance to account for muscles with very low stimulation amplitude.

For the second step, we solved another optimal control problem that optimized stimulation amplitude and timing for these two new muscles (Table 2, Treatment Optimization Problem 2.5). The problem formulation was identical to that of the second FastFES treatment scenario except that the two stimulated muscles were changed, and thus stimulation amplitude, on-time, and duration for both muscles were again the main optimization parameters. This optimization was formulated to investigate whether stimulation of two different muscles in place of the standard ones might be a better choice for this particular patient.

## Results

### Neuromusculoskeletal Model Personalization

**Figure 2:**
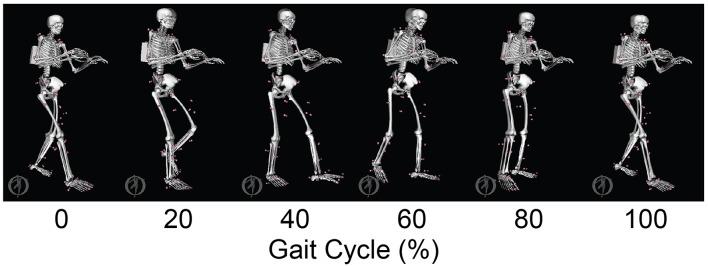
Animation strip comparing the subject's experimental gait motion (translucent skeleton) with his verification gait motion (opaque skeleton). The verification gait motion was predicted by a direct collocation optimal control problem that used the subject's personalized neuromusculoskeletal model but did not track any experimental quantities in the cost function. This gait motion prediction was used to gain confidence in the personalized model and optimal control problem formulation.

The neuromusculoskeletal model personalization process successfully calibrated model parameter values to closely reproduce the subject's marker motion, joint motion, joint moment, ground reaction, and muscle activation data. EMG-driven joint moments from muscle-tendon model personalization matched inverse dynamic joint moments with root-mean-square errors (RMSE) ranging from 2.5 to 6.4 Nm and mean absolute errors (MAE) between 2.0 and 4.7 Nm (Table 3). Ground reaction forces and moments from ground contact model personalization reproduced experimental ground reactions with RMS errors below 2.1 N for forces and 2.9 Nm for moments (Table 4). The verification optimal control problem using the complete personalized model produced a dynamically consistent full-body walking motion that matched lower body inverse kinematic joint angles to within 3.2 deg RMSE and 2.2 deg MAE, lower body inverse dynamic joint moments to within 4.8 Nm RMSE and 3.4 Nm MAE, measured ground reaction forces to within 31 N RMSE and 17 N MAE, and calibrated muscle activations to within 0.05 RMSE and 0.04 MAE (Figure 2; Table 5).

**Table 3:** Root-mean-square error (RMSE), mean absolute error (MAE), maximum absolute error (MaxAE), and range in joint moments over the gait cycle from muscle-tendon model personalization.

**Quantity**	**Hip extension (Nm)**	**Hip abduction (Nm)**	**Knee extension (Nm)**	**Ankle plantarflexion (Nm)**	**Ankle eversion (Nm)**
RMSE	5.37	6.19	4.90	6.36	2.54
MAE	4.16	4.69	3.81	4.62	1.98
MaxAE	21.59	30.28	21.92	42.05	12.17
Range	98.69	86.98	69.86	151.87	31.34

Errors represent the difference between inverse dynamic joint moments calculated by OpenSim and net joint moments calculated by the calibrated EMG-driven model. Quantities represent averages between the two legs for 80 gait cycles (40 per leg) used in the model personalization process.

**Table 4:** Root-mean-square error (RMSE), mean absolute error (MAE), maximum absolute error (MaxAE), and range in ground reaction forces and moments over the gait cycle from foot-ground contact model personalization.

**Quantity**	**Anterior force (N)**	**Superior force (N)**	**Lateral force (N)**	**Anterior moment (Nm)**	**Superior moment (Nm)**	**Lateral moment (Nm)**
RMSE	1.79	2.06	1.64	2.89	0.70	2.34
MAE	1.47	1.54	1.33	2.31	0.60	1.97
MaxAE	4.77	6.22	4.21	5.78	1.29	5.65
Range	160.47	770.68	59.54	23.73	14.91	71.73

Errors represent the difference between ground reactions measured experimentally and ground reactions calculated by calibrated two-segment foot-ground contact models. Quantities represent averages between the two legs for representative gait cycle.

**Table 5:** Root-mean-square error (RMSE), mean absolute error (MAE), maximum absolute error (MaxAE), and range in joint angles, joint moments, ground reaction forces, and muscle activations from the verification optimal control problem.

**General quantity**	**Specific quantity**	**RMSE**	**MAE**	**MaxAE**	**Range**
Joint angles (deg)	Hip flexion	2.3	1.8	5.2	34.0
	Hip adduction	1.4	1.1	2.8	13.6
	Knee flexion	3.2	2.2	8.5	65.0
	Ankle dorsiflexion	1.4	0.8	4.7	26.9
	Ankle inversion	1.8	1.4	4.6	15.4
Joint moments (Nm)	Hip extension	3.5	2.8	10.2	63.8
	Hip abduction	4.8	3.4	12.9	61.5
	Knee extension	1.9	1.3	7.5	41.0
	Ankle plantarflexion	3.8	2.2	12.4	107.0
	Ankle eversion	1.4	1.0	4.4	20.1
Ground reaction forces (N)	Normal	30.9	16.7	119.1	782.7
	Propulsive	6.7	4.4	24.9	159.6
	Lateral	14.1	10.3	30.8	68.9
Muscle activations (unitless)	Uniarticular hip	0.023	0.015	0.121	0.749
	Uniarticular knee	0.033	0.025	0.112	0.440
	Uniarticular ankle	0.044	0.033	0.141	0.834
	Biarticular hip-knee	0.023	0.020	0.091	0.353
	Biarticular knee-ankle	0.016	0.013	0.036	0.155

Errors represent the difference between quantities measured experimentally or calculated from experimental data and quantities predicted by the verification problem. None of the quantities included in this table was tracked in the verification cost function. Quantities represent averages between the two legs for the representative gait cycle.

### FastFES Treatment Optimization

**Figure 3:**
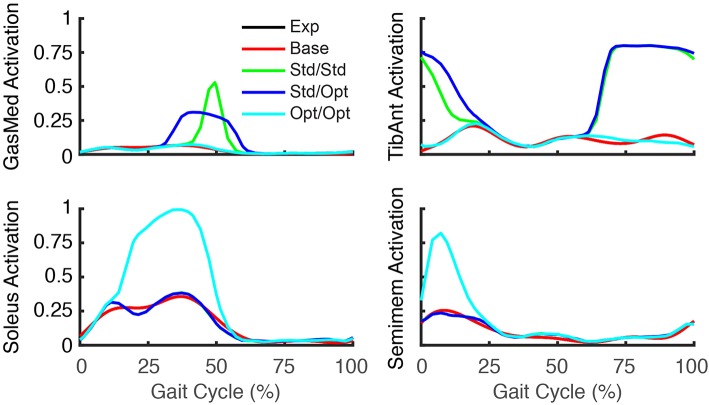
Experimental and predicted activation patterns for electrically stimulated muscles. Activation patterns for standard muscle selection involving stimulation of GasMed and TibAnt (top row) and optimal muscle selection involving stimulation of Sol and Semimem (bottom row) are presented for the paretic leg. Exp indicates experimental curves, Base indicates curves from the baseline treatment optimization with no muscle stimulation, Std/Std indicates curves from the FastFES treatment optimization using standard muscle selection with standard stimulation timing, Std/Opt indicates curves from the FastFES treatment optimization using standard muscle selection with optimized stimulation timing, and Opt/Opt indicates curves from the FastFES treatment optimization using optimized muscle selection with optimized stimulation timing.

The three FastFES treatment optimizations predicted progressively lower AP force asymmetry relative to the baseline optimization with no muscle stimulation (Table 6) along with visible changes in muscle activation patterns relative to baseline (Figure 3). Stimulation of standard muscles with standard timing decreased the difference in AP force impulse between the two legs by 41% relative to baseline, stimulation of standard muscles with optimal timing produced a 45% decrease relative to baseline, and stimulation of optimal muscles with optimal timing yielded a 64% decrease. When stimulation timing was allowed to change for standard muscle selection, TibAnt stimulation amplitude and timing remained relatively unchanged, while GasMed stimulation amplitude was decreased by 66% and stimulation duration was increased by 600% to cover a much larger portion of stance phase with greater similarity to healthy stimulation timing (Table 7). When the two stimulated muscles were allowed to change, the preliminary optimization selected soleus (Sol) and semimembranosus (Semimem) as the best two muscles to stimulate, and the subsequent treatment optimization predicted unique stimulation timings present only during stance phase (Table 7).

**Table 6:** Difference in anterior-posterior (AP) force impulse between the two legs for the baseline optimization with no muscle stimulation and the three FastFES treatment optimizations, along with percent reduction in AP force impulse difference relative to baseline.

**Treatment optimization problem**	**AP impulse difference (Ns)**	**Reduction in difference (%)**
No stimulation-baseline	19.5	—
Stimulate standard muscles with standard timing	11.6	40.6
Stimulate standard muscles with optimal timing	10.6	45.4
Stimulate optimal muscles with optimal timing	7.0	64.1

**Table 7:** Muscle stimulation parameters found by FastFES treatment optimizations.

**Treatment optimization problem**	**Stimulated muscles**	***A***	***t*_**on**_ (%)**	***t*_**off**_ (%)**
Stimulate standard muscles with standard timing	GasMed	0.70	46	49
	TibAnt	0.70	65	2
Stimulate standard muscles with optimal timing	GasMed	0.24	34	55
	TibAnt	0.70	64	6
Stimulate optimal muscles with optimal timing	Sol	0.62	16	45
	Semimem	0.70	0	7

Muscle name abbreviations are defined in Table 1. A is stimulation amplitude, ton is stimulation on-time as percent of gait cycle, and toff is stimulation off-time as percent of gait cycle. Off-time is less than on-time if the stimulation wrapped around the end of the gait cycle to the start of the same gait cycle.

**Figure 4:**
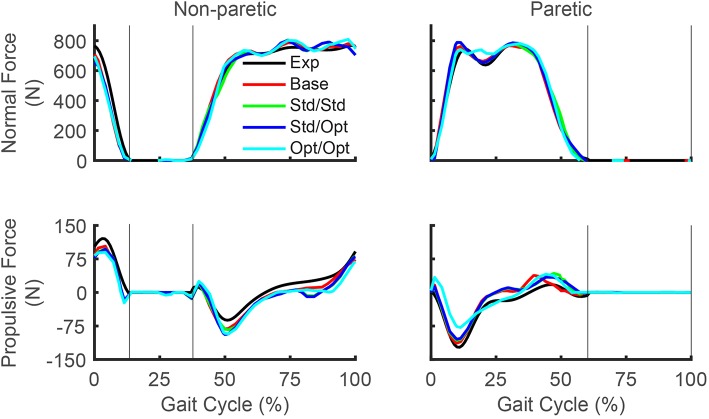
Experimental and predicted ground reaction forces over the gait cycle. Normal ground reaction force (top row) and propulsive ground reaction force (bottom row) are presented for the non-paretic leg (left column) and the paretic leg (right column). Thin vertical lines indicate locations of heel strike and toe off. Exp indicates experimental curves, Base indicates curves from the baseline treatment optimization with no muscle stimulation, Std/Std indicates curves from the FastFES treatment optimization using standard muscle selection with standard stimulation timing, Std/Opt indicates curves from the FastFES treatment optimization using standard muscle selection with optimized stimulation timing, and Opt/Opt indicates curves from the FastFES treatment optimization using optimized muscle selection with optimized stimulation timing. Note that the non-paretic leg is the left leg while the paretic leg is the right leg.

Predicted reductions in AP force asymmetry were accompanied by notable changes in predicted propulsive as well as braking force (Figure 4; Table 8). Experimentally, more propulsive force (positive peak and impulse) and less braking force (negative peak and impulse) were present on the non-paretic (left) side than on the paretic (right) side. Each of the three FastFES treatment optimizations decreased propulsive force and increased braking force on the non-paretic side while simultaneously increasing propulsive force and decreasing braking force on the paretic side. The one exception was standard muscles with optimal timing, which predicted a decreased propulsive force peak with an increased propulsive force impulse on the paretic side. Overall, the extent of predicted propulsive force changes tended to increase with each subsequent treatment optimization, though this general trend was not strictly followed. The AP force profile was the most similar between the two sides for optimal muscle selection with optimal stimulation timing.

**Table 8:** Peak and impulse of propulsive force and breaking force for the paretic and non-paretic leg for the baseline optimization with no muscle stimulation and the three FastFES treatment optimizations, along with percent reductions relative to baseline (indicated in parentheses).

**Force**	**Treatment optimization problem**	**Paretic leg**		**Non-paretic leg**	
		**Peak (N)**	**Impulse (Ns)**	**Peak (N)**	**Impulse (Ns)**
Propulsive	No stimulation—baseline	37.8 (–)	7.2 (–)	106.2 (–)	25.6 (–)
	Stimulate standard muscles with standard timing	44.9 (18.6%)	9.9 (36.8%)	97.7 (−8.0%)	22.6 (−11.6%)
	Stimulate standard muscles with optimal timing	34.7 (−8.3%)	9.5 (31.9%)	97.0 (−8.6%)	22.7 (−11.3%)
	Stimulate optimal muscles with optimal timing	41.6 (9.9%)	10.1 (39.0%)	91.1 (−14.2%)	20.0 (−21.9%)
Braking	No stimulation—baseline	−113.3 (–)	−22.0 (–)	−81.9 (–)	−20.9 (–)
	Stimulate standard muscles with standard timing	−107.2 (−6.1%)	−21.3 (−3.3%)	−83.9 (2.5%)	−22.4 (7.4%)
	Stimulate standard muscles with optimal timing	−104.5 (−8.8%)	−20.4 (−7.3%)	−94.2 (15.0%)	−22.9 (9.7%)
	Stimulate optimal muscles with optimal timing	−78.8 (−34.5%)	−18.7 (−15.0%)	−92.7 (13.2%)	−21.7 (3.7%)

**Figure 5:**
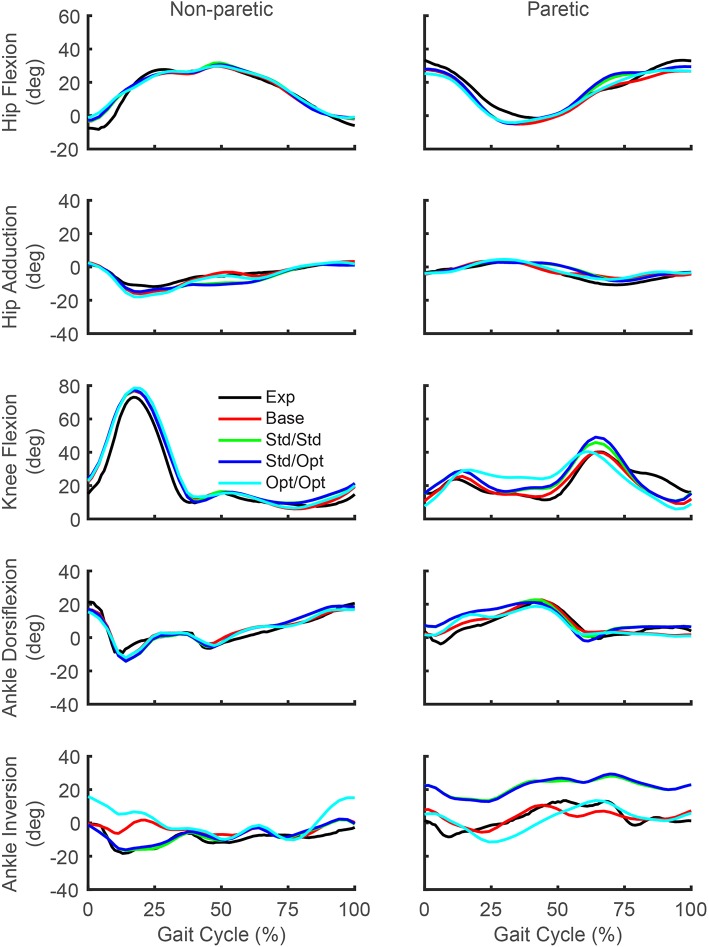
Experimental and predicted lower body joint angles over the gait cycle. Hip flexion (first row), hip adduction (second row), knee flexion (third row), ankle dorsiflexion (fourth row), and ankle inversion (fifth row) are presented for the non-paretic leg (left column) and the paretic leg (right column). The legend is the same as in Figure 2.

While the predicted joint angles for the four treatment optimizations were similar to the experimental gait motion, some differences were still evident (Figure 5, see animations in Supplementary Material). For paretic leg hip flexion relative to the experimental trajectory, all four optimizations predicted a decrease over most of stance phase, an increase during the first half of swing phase, and a decrease during the second half of swing phase. These changes made the paretic leg hip flexion trajectories more similar to the non-paretic leg hip flexion trajectories. For paretic leg knee flexion, the four optimizations predicted an increase over most of stance phase and a decrease at the end of swing phase. The increase during stance phase was most pronounced for the optimization that used optimal muscle selection. The decrease at the end of swing phase made the paretic leg knee flexion trajectories more similar to the non-paretic leg knee flexion trajectories. Maximum paretic leg knee flexion just after toe off increased only for the two optimizations that used standard muscle selection, and even then, the increases did not approach the corresponding peak values on the non-paretic side. For paretic leg ankle dorsiflexion, all four optimizations predicted an increase over the first half of stance phase. For paretic leg ankle inversion, the two optimizations that used standard muscle selection predicted an increase over the entire gait cycle, while the optimization that used optimal muscle selection predicted a decrease over most of stance phase. The same optimization predicted an increase in non-paretic leg ankle inversion over much of the gait cycle.

**Figure 6:**
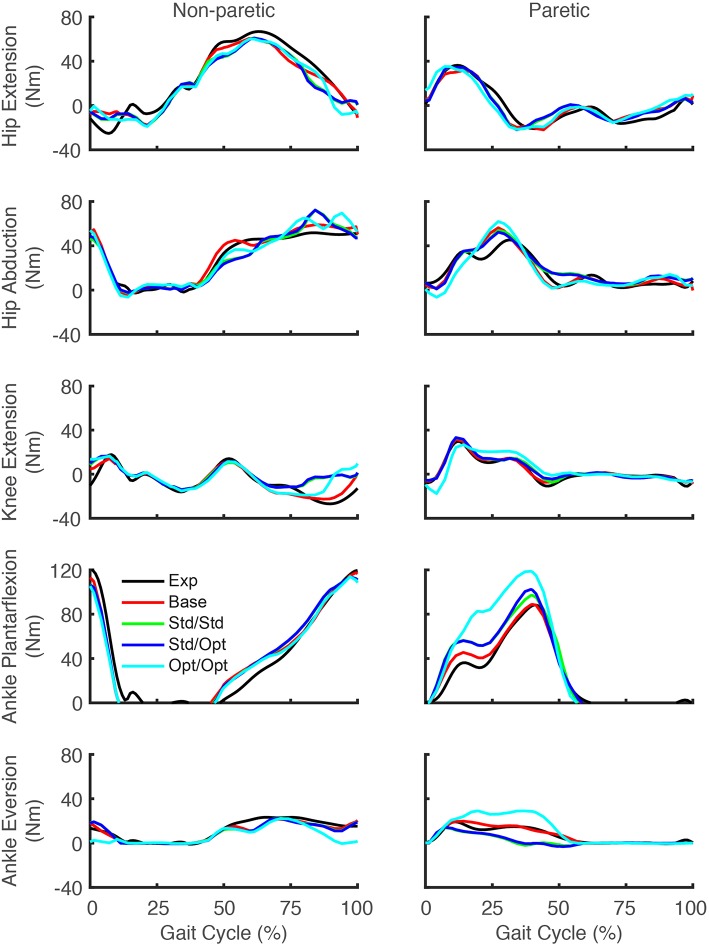
Experimental and predicted lower body joint moments over the gait cycle. Hip extension moment (first row), hip abduction moment (second row), knee extension moment (third row), ankle plantarflexion moment (fourth row), and ankle eversion moment (fifth row) are presented for the non-paretic leg (left column) and the paretic leg (right column). The legend is the same as in Figure 2.

Predicted lower body joint moments also exhibited notable changes in response to the different simulated stimulation conditions (Figure 6). For all four optimizations, the hip extension and abduction moments were similar to experimental trajectories, with the largest deviation being an increased paretic leg hip abduction moment in the middle of stance phase. The knee extension moment on the paretic side showed little change from the experimental trajectory except for the optimization that used optimal muscle selection, which predicted a decreased knee extension moment at the start of stance phase and an increased moment from the middle to the end of stance phase. On the non-paretic side, the knee extension moment increased at the end of stance phase for all four optimizations. The paretic ankle plantarflexion moment exhibited the most prominent joint moment changes, with all four optimizations predicting an increase over stance phase relative to the experimental trajectory. The optimization that used optimal muscle selection predicted the largest increase, with the peak value reaching the corresponding peak on the non-paretic side. The paretic ankle eversion moment also exhibited prominent changes. The two optimizations that used standard muscle selection predicted a decreased ankle eversion moment over most of stance phase, while the optimization that used optimal muscle selection predicted an increase over the same region.

## Discussion

This study used a personalized neuromusculoskeletal walking model coupled with direct collocation optimal control to predict how FastFES treatments should be implemented to maximize propulsive force symmetry for an individual post-stroke who was a non-responder to the standard FastFES training protocol. Though FastFES is a promising treatment for post-stroke gait neurorehabilitation, methods for customizing FastFES prescriptions to the unique needs of individual patients have yet to be developed. Using treadmill gait data collected from a non-responder to FastFES training, we personalized a full-body neuromusculoskeletal model and then used it to predict improvement in the subject's AP force symmetry for three FastFES treatment scenarios: (1) Standard muscle selection with standard stimulation timing, (2) Standard muscle selection with optimized stimulation timing, and (3) Optimized muscle selection with optimized stimulation timing. Overall, the more flexibility a FastFES treatment optimization was given, the more the subject's predicted AP force asymmetry was reduced. Our results suggest that for this particular subject, (1) Stimulation of standard muscles (i.e., TibAnt and GasMed) with standard timing should produce an acute improvement in the subject's propulsive force symmetry between the two legs, (2) A comparable improvement in propulsive force symmetry could potentially be achieved for this subject by stimulating TibAnt with standard settings and GasMed with decreased amplitude but increased duration, (3) A larger improvement in the subject's propulsive force symmetry could potentially be achieved by stimulating Sol and Semimem in place of TibAnt and GasMed, and (4) Large improvements in propulsive force symmetry may not guarantee large improvements in joint motion symmetry. Thus, future optimal control studies should explore adding kinematic symmetry terms to the optimization cost function so that improvements in both types of symmetry can be predicted simultaneously. The methodology developed in this study therefore provides only a first step toward computational design of personalized FastFES prescriptions that are customized to the unique functional limitations of the patient.

Since our subject was the non-responder in a recent FastFES clinical study (Allen et al., 2018), an important question is why our treatment optimization using standard muscle selection with standard stimulation timing predicted a large improvement in propulsive force symmetry. This apparent inconsistency can be explained by considering the differences between these two situations. During training in the laboratory *with stimulation* (the situation predicted by the model), AP force symmetry is improved due to an acute response to the electrical stimulation, often termed an orthotic effect. This orthotic effect demonstrates that the FES is able to augment force generation in the ankle muscles and generate greater paretic leg propulsion while the FES is on. In contrast, after multiple sessions of FastFES training, when gait performance is evaluated in the community or measured in the lab *without stimulation*, AP force symmetry is determined by the therapeutic or long-term retention effect of the treatment. The therapeutic effect may be influenced by multiple factors including the magnitude of favorable neuroplasticity induced through repeated training as well as improvements in muscle strength, cardiovascular endurance, and psychosocial factors. EMG data published in the previous FastFES clinical study (Allen et al., 2018) indicates that our subject exhibited statistically significant increases in unstimulated Sol and TibAnt activity pre- to post-training. However, increases in Sol activity were small in magnitude and thus potentially insignificant functionally, while the increase in TibAnt activity was in stance phase rather than swing phase as desired. Thus, our results suggest that for this subject, the level of acute improvement obtained *due to stimulation* during training may have been larger than the level of long-term improvement obtained *due to neuroplasticity* after the completion of training. The fact that this subject improved AP force symmetry during stimulation but did not retain the improvement afterward when tested without stimulation explains in part why he was classified as a non-responder to the intervention. A challenge for the future is finding a way to predict reliably which muscle excitations are the most amenable to long-term training-induced neuroplasticity.

Three additional considerations may help explain this apparent inconsistency further. First, the subject did improve peak paretic propulsive force following FastFES training (Allen et al., 2018), but he started with extremely low peak propulsive force (as seen in Figure 4) and achieved only a small improvement following training. Second, our optimal control problems quantified improvements in propulsive force symmetry using the integral of AP force over the gait cycle, which accounts for not only peak propulsive force but also peak braking force using a mathematical function that is continuous and differentiable. In the clinical study, the change in the subject's peak paretic braking force was not reported, though our optimal control predictions suggest that standard muscle selection may not reduce peak paretic braking force as substantially as does optimal muscle selection (Figure 4; Table 8). Third, it is possible that the subject did not try to minimize propulsive force asymmetry when relearning to walk. A potentially insightful experiment would be to provide the subject with real-time feedback of his propulsive force asymmetry and instruct him to attempt to minimize it, similar to recent studies performed on healthy individuals (Schenck and Kesar, 2017) and individuals post-stroke (Genthe et al., 2018). Such an experiment could elucidate whether the subject's propulsive force asymmetry is primarily due to neural control limitations, biomechanical constraints, or a subconscious decision to optimize other quantities (e.g., metabolic cost Zarrugh et al., 1974; Bertram, 2005).

Our optimal control predictions suggest two alternate FastFES protocols that could potentially benefit this subject. The first alternate protocol would decrease GasMed's stimulation amplitude while prolonging its stimulation duration. This change is consistent with GasMed being stimulated to increase late-stance paretic propulsion, whereas TibAnt is stimulated to prevent foot drop during swing phase (Hakansson et al., 2011; Kesar et al., 2011). This protocol has the potential benefits of reducing GasMed fatigue and stimulation discomfort while also reducing the sensitivity of the resulting motion to the selected stimulation on-time and off-time. The second alternate protocol would replace GasMed and TibAnt stimulation with Sol and Semimem stimulation. This protocol has never been investigated, so it is unknown whether stimulation of these alternate muscles would facilitate or hinder the subject's long-term neuroplasticity and motor learning. A potential benefit of the Sol and Semimem stimulation protocol is that it may produce a large decrease in braking force peak and impulse for the paretic leg (see Figure 4; Table 8). For any FastFES protocol change, implementation of predicted stimulation amplitudes would be a challenge. Some method would be needed to calibrate the relationship between model-predicted and experimentally-applied stimulation amplitude (Kesar et al., 2008; Perumal et al., 2008). However, if the predicted relative stimulation amplitude between the two stimulated muscles was reliable, then it could be possible to constrain the two stimulation amplitudes to maintain the desired ratio. With this approach, only a single stimulation amplitude would need to be manipulated experimentally when exploring subject-specific stimulation settings, realizing that the maximum achievable stimulation will depend on the subject's tolerance of the discomfort caused by electrical stimulation.

It is interesting to consider whether the two muscles (Sol and Semimem) selected by our third treatment optimization would be logical choices based on interpretation of their biomechanical roles. While paretic (right) propulsive force generation was clearly inhibited in the subject's experimental gait pattern, braking force was also larger on the paretic side than on the non-paretic side, likely due to poor coordination. Thus, muscle stimulation that acts to decrease early-stance braking and/or increase late-stance propulsion would improve propulsion symmetry in this subject. Indeed, the optimization that selected the best two muscles to stimulate chose one muscle to increase propulsive force in late stance (Sol) and another muscle to decrease braking force in early stance (Semimem), consistent with minimization of AP force impulse asymmetry. Several studies have reported that Sol and GasMed contribute to forward acceleration of the trunk in mid to late stance (Neptune et al., 2001; Liu et al., 2006) and that both contribute to propulsive ground reaction force in late stance (Neptune et al., 2004; Allen and Neptune, 2012). Thus, selection of Sol as a replacement for GasMed is not surprising. Although stimulating Sol can improve propulsive force in late-stance, stimulation of Sol during mid-stance may actually contribute to *increased* braking forces (Neptune et al., 2004). In contrast, published studies have also reported that the hamstrings contribute to propulsive ground reaction force in early to mid-stance (Neptune et al., 2004; Allen and Neptune, 2012), potentially explaining the selection of Semimem to counteract increased braking from the Sol. However, these choices also resulted in increased knee flexion throughout most of stance phase, which may not be desirable from either a metabolic perspective or an aesthetic perspective. Finally, despite elimination of TibAnt stimulation, our optimal treatment still predicted that the paretic toe would clear the ground, potentially through increased knee flexion at the start of swing phase (Figure 5).

Though muscle synergy analysis is often used to quantify control complexity and inter-muscle coupling in experimentally measured EMG signals, our study used muscle synergy concepts for broader control-related reasons. First, we used a low-dimensional set of synergy activations rather than 36 independent muscle activations to control each leg since synergy activations have been shown to generate more accurate predictions of walking under new conditions (Meyer et al., 2016). Second, we used synergy rather than muscle activation controls to simplify the model's control structure, which significantly improves computational speed and convergence of optimal control walking predictions (Meyer et al., 2016). Third, we used synergy activation controls so that missing muscle activations could be predicted as linear combinations of the synergy activations extracted from muscle activations with associated EMG measurements (Bianco et al., 2017).

While our choice of five synergies to control each leg was based on achieving at least 95% VAF for each individual muscle activation in both legs, this choice was informed by three additional considerations. First, to closely match all available experimental data (i.e., joint angles, ground reactions, and muscle activations), more synergies are required than indicated by synergy analysis of muscle activation data alone. Because our walking predictions are dynamically consistent, the muscle activations controlling the model must be of high enough fidelity to reproduce the subject's experimental data closely. Based on our previous work (Meyer et al., 2016), the number of synergies found by synergy analysis of EMG data alone may not be enough to produce a simulated walking motion that tracks experimental data as closely as desired. The reason is that matching joint motion, ground reaction, and EMG data simultaneously with muscle synergy controls is a much more constrained situation than matching only EMG data with muscle synergy controls, thereby necessitating a larger number of synergies than would have been retained otherwise. Second, to predict missing muscle activations using synergy activations extracted from muscles with EMG data, more synergies are required than indicated by synergy analysis of EMG data alone (Bianco et al., 2017). As noted earlier, 95% total VAF does not guarantee a comparably high individual muscle %VAF. To minimize the risk of poor construction of missing activations, we chose the number of synergies so that all measured muscle activations were reconstructed with at least 95% VAF. Use of more than 5 synergies would likely not improve our ability to fit measured activations or predict missing activations, while use of fewer than 5 synergies would produce poorer fitting of some measured activations and likely poorer prediction of missing activations.

**Figure 7:**
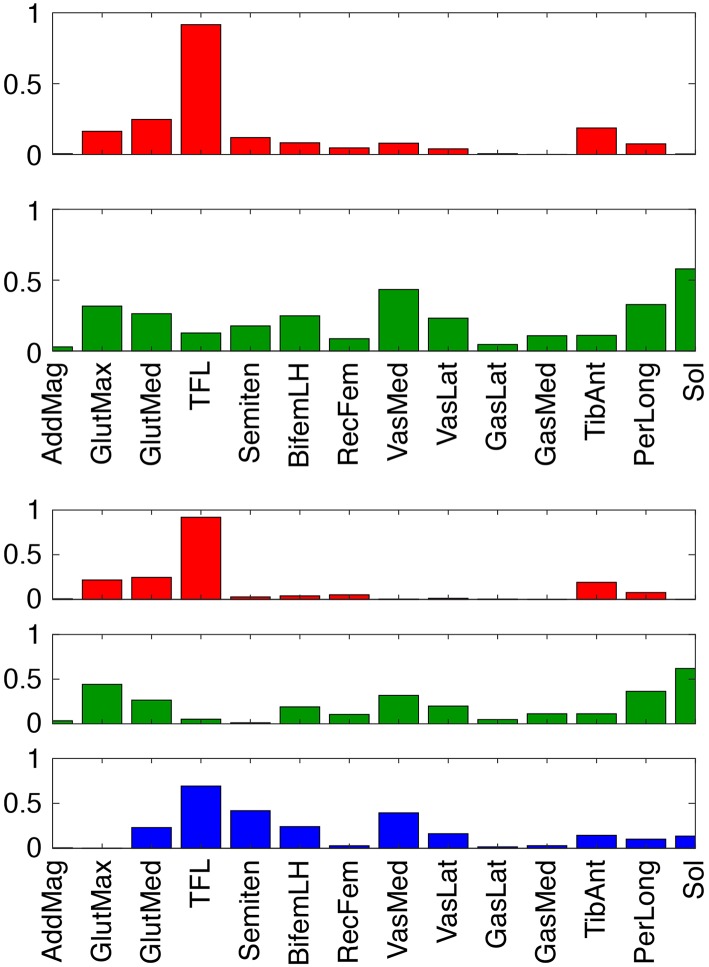
Synergy vectors for 14 paretic leg muscle activations derived from measured EMG signals for 2 (top) and 3 (bottom) synergies. Prior to synergy analysis, measured EMG signals were processed and normalized as part of the muscle-tendon model personalization process. Muscle name abbreviations are listed in Table 1.

To evaluate theoretically whether stimulation of our two predicted muscles (Sol and Semimem) might be more effective for this subject than stimulation of the two standard muscles (GasMed and TibAnt), we examined the structure of the subject's synergy vectors (SVs) for 2 and 3 paretic leg synergies (Figure 7). We chose these low numbers of synergies since they achieved 95% total VAF for experimental muscle activations from both legs and provide the simplest perspective for interpretation purposes. For the 2-synergy solution, paretic GasMed did not appear predominantly in either SV, while paretic TibAnt appeared with moderate weight in the first SV. Thus, if training does not alter the composition of the SVs, then increased recruitment of GasMed may produce large unwarned increases in the recruitment of other muscles. In contrast, paretic Sol possessed the largest weight in the second SV, and paretic Semimem possessed a moderate weight in the same SV. Thus, if stimulating paretic Sol and Semimem during FastFES training resulted in enhanced recruitment of the second synergy, the activations of both muscles would increase together, which would be undesirable since no overlap exists in the predicted optimal stimulation timing for these two muscles. Not surprisingly, this interpretation changes for the 3-synergy solution, where paretic Sol and Semimem appear prominently in two different SVs—the second synergy for Sol and the third synergy for Semimem. Since the optimal stimulation timing for these two muscles is non-overlapping, decoupling the recruitment of these two muscles could be beneficial. Increased recruitment of the second and third synergies would also increase recruitment of other muscles (e.g., gluteus maximus, peroneus longus, tensor fascia latae, and vastus medialis), which may or may not be beneficial. In contrast, neither GasMed nor TibAnt appears prominently in any of the three SVs, suggesting that it could be difficult to increase the activation of these muscles without creating undesirable activation increases in other muscles. If the number of synergies is increased further, GasMed and TibAnt remain weakly represented in the SVs, while Sol and Semimem become more dominant in separate SVs, which is consistent with the idea that the activation of these two muscles could potentially be trained independently. Thus, analysis of the subject's muscle synergies at least hints at the possibility that the two new muscles selected by the optimization may be worth considering for stimulation.

Interestingly, the three FastFES treatment optimizations predicted comparable values for peak paretic propulsive force during stance phase (Figure 4) as well as peak paretic knee flexion during swing phase (Figure 5). In both cases, these peak values were substantially lower than the corresponding peak values predicted for the non-paretic leg. For our subject, a low value of peak paretic propulsive force resulted in a slow self-selected walking speed, while a low value of peak paretic knee flexion necessitated a compensatory hip hiking strategy to ensure that the paretic foot cleared the ground during swing phase (Chen et al., 2005). Since AP force asymmetry was minimized in the cost function, these observations suggest that the personalized model hit a “ceiling” on the increase in paretic propulsive force achievable using electrical stimulation of only two muscles. While the model may have also hit a “ceiling” on the achievable increase in paretic knee flexion, this conclusion is less clear since a knee flexion asymmetry term was not included in the cost function. If such a term were added, the resulting increase in paretic knee flexion would likely be accompanied by a corresponding decrease in paretic propulsive force. To improve peak paretic propulsive force and peak paretic knee flexion to the desired levels simultaneously, the optimal control problem would need to change the subject's paretic leg muscle synergies. A future optimal control study could therefore explore finding the smallest changes to a single paretic leg synergy activation that would bring the subject's peak paretic propulsive force and peak paretic knee flexion as close as possible to their desired levels. The predicted synergy changes could then inform complementary neurorehabilitation efforts or even electrical stimulation of the spinal cord to recruit the identified synergy (Wenger et al., 2016).

Our study possesses several important limitations that inform interpretation of our current results and suggest directions for future investigation. First, only a single subject was studied. We specifically selected a non-responder to FastFES training so as to maximize our chances of identifying alternate stimulation protocols that could potentially improve the subject's walking ability. Whether or not the same modeling approach would work for other subjects will require further investigation. Second, no measurements were available for the forces and torques exerted by the handlebar on each hand, plus the hand loads estimated during the model personalization process were applied to the model during the treatment optimization process. Experimental measurement of hand loads would greatly simplify model personalization. Allowing the hand loads to vary during treatment optimization could alter our propulsive force predictions. Third, experimental stimulation does not target individual muscles as directly as modeled in this study. In practice, medial and lateral gastrocnemius are often stimulated together by a single electrode. Even if GasMed and TibAnt were well-targeted for electrical stimulation, some stimulation would likely “bleed” into other plantarflexor and dorsiflexor muscles. Modeling of this “bleeding” phenomenon would impact our treatment predictions, though the extent to which the predictions would be changed is unknown. Fourth, no measure of kinematic asymmetry was included in the optimal control cost function. When initiating this study, we expected that improved propulsive force symmetry would naturally result in improved joint motion symmetry. This expectation proved to be incorrect, suggesting that some measure of kinematic asymmetry should be included in future optimal control studies of FastFES treatment design. However, no published study to date has presented an optimal control problem formulation that is capable of turning an asymmetric walking motion into a symmetric one. Thus, development of a problem formulation that enforces kinematic symmetry remains an important challenge for the neuromusculoskeletal modeling research community. Fifth, simulated electrical stimulation was explored for only two paretic leg muscles due to current technical limitations in the FastFES hardware and software. If stimulation of three or even four muscles was investigated, it is possible that substantial improvements in both propulsive force symmetry and kinematic symmetry could be predicted. However, stimulation of more than two muscles is not feasible with the electrical stimulation system used in this study. Sixth, our FastFES treatment optimizations assumed that the subject's neural control strategy remained largely unchanged by the application of FES. In reality, if a subject responds to FastFES training, one would expect his or her neural control strategy to change over the course of treatment as favorable neuroplasticity occurred (Allen et al., 2018). Modeling how a patient's neural control strategy changes over time with training would likely alter our treatment predictions.

Another important limitation of this study was the amount of time and effort required to perform the entire sequence of optimizations used for model personalization and treatment optimization. Generation of the results reported in this study required over 2 years of effort by a single Ph.D. student. Once the entire process was set up for a new subject performing a new task (walking with hands on handlebars), all model personalization optimizations could be completed in roughly 5 h of CPU time, while each treatment optimization required between 3 and 15 min of CPU time. Thus, the primary bottleneck was not computation time but rather the time required to learn the entire computational workflow, process the experimental data to get them into the correct format, and identify appropriate optimization problem formulations to get each step to work properly the first time. In the present study, the most challenging problem formulation issues were related to how to minimize propulsive force asymmetry, model the hands grasping the handlebar, and predict the best two muscles to stimulate. None of these issues had been explored in previous optimal control studies of human walking, and all of them required running hundreds of optimal control problems before appropriate problem formulations could be identified. We are continuing to refine our model personalization and treatment optimization workflow so that the steep learning curve currently required to become proficient in the entire process can be eliminated as a bottleneck.

One of the biggest limitations of this study was our inability to evaluate experimentally our optimal FastFES treatment prediction. Such an evaluation would have required completion of an additional training study for this subject, which unfortunately we were unable to perform. We hope to be able to apply our optimal treatment prediction to this subject, as well as explore the use of model-based optimal treatment predictions for other subjects, as part of a future FastFES training study.

In conclusion, this study explored the feasibility of using subject-specific neuromusculoskeletal models combined with direct collocation optimal control to predict novel FastFES treatment prescriptions that may improve a specified treatment target—in this case, inter-leg propulsive force symmetry. The ability to tailor neurorehabilitation treatments to the unique needs of individual patients would be an important next step in modern healthcare. In the case of stroke, walking deficits vary widely from patient to patient, highlighting the need for an objective and effective treatment customization process. In the current study, a computational approach to FastFES treatment customization predicted that changing the stimulation amplitude and timing of a typically stimulated muscle, or changing which two muscles are stimulated, may improve a specific subject's paretic propulsion significantly. While this computational approach is still too difficult and time consuming to be feasible on a large scale, future improvements in computational methodology and technology may eventually make it possible to perform this approach on a routine basis, potentially allowing treatment decisions to be based on objective predictions of a patient's post-treatment function.

## Data Availability Statement

The Matlab code and experimental data used to perform this study can be found on Simtk.org at https://simtk.org/projects/predictfastfes.

## Ethics Statement

The human subject was recruited at Emory University and gave informed written consent. The study was approved by the institutional review boards of Emory University where the data were collected and the University of Florida where the data were modeled.

## Author Contributions

NS performed all model personalization and treatment optimization tasks, wrote the first draft of the manuscript, and assisted with preparation of figures and tables. AM assisted with development of the personalized neuromusculoskeletal model, modification of the optimal control problem formulations to accommodate the goals of the present study, and review of the manuscript draft. JA oversaw experimental data collection for the project, processed all experimental data, and provided feedback on the manuscript draft. LT and TK recruited the experimental subject, organized the experimental data collection session, helped identify treatment scenarios to optimize, and provided feedback on the manuscript draft. BF coordinated the entire project, oversaw model personalization and treatment optimization tasks, edited the manuscript draft, and prepared the final figures and tables.

### Conflict of Interest

The authors declare that the research was conducted in the absence of any commercial or financial relationships that could be construed as a potential conflict of interest.

## Supplementary Material

Supplementary Video 1 OpenSim animations comparing experimental walking motion (translucent skeleton) with predicted walking motions (opaque skeletons) for baseline treatment optimization.

Supplementary Video 2 FastFES treatment optimizations using standard muscle selection with standard stimulation timing.

Supplementary Video 3 Standard muscle selection with optimized stimulation timing.

Supplementary Video 4 Optimized muscle selection with optimized stimulation timing.

## References

[B1] Abellan van KanG.RollandY.AndrieuS.BauerJ.BeauchetO.BonnefoyM.. (2009). Gait speed at usual pace as a predictor of adverse outcomes in community-dwelling older people an International Academy on Nutrition and Aging (IANA) task force. J. Nutr. Health Aging 13, 881–889. 10.1007/s12603-009-0246-z19924348

[B2] Allen J. L. Neptune R. R. (2012). Three-dimensional modular control of human walking. J. Biomech. 45, 2157–2163. 10.1016/j.jbiomech.2012.05.037 22727468 PMC3405171

[B3] Allen J. L. Ting L. H. Kesar T. M. (2018). Gait rehabilitation using functional electrical stimulation induces changes in ankle muscle coordination in stroke survivors: a preliminary study. Front. Neurol. 9:1127. 10.3389/fneur.2018.01127 30619077 PMC6306420

[B4] Awad L. N. Reisman D. S. Kesar T. M. Binder-Macleod S. A. (2014). Targeting paretic propulsion to improve post-stroke walking function: a preliminary study. Arch. Phys. Med. Rehabil. 95, 840–848. 10.1016/j.apmr.2013.12.012 24378803 PMC4160043

[B5] Awad L. N. Reisman D. S. Pohlig R. T. Binder-Macleod S. A. (2016). Reducing the cost of transport and increasing walking distance after stroke: a randomized controlled trial on fast locomotor training combined with functional electrical stimulation. Neurorehabil. Neural Repair 30, 661–670. 10.1177/1545968315619696 26621366 PMC4885807

[B6] Azevedo Coste C. Jovic J. Pissard-Gibollet R. Froger J. (2014). Continuous gait cycle index estimation for electrical stimulation assisted foot drop correction. J. Neuroeng. Rehabil. 11:118. 10.1186/1743-0003-11-118 25108539 PMC4266978

[B7] Balaban B. Tok F. Yavuz F. Yaşar E. Alaca R. (2011). Early rehabilitation outcome in patients with middle cerebral artery stroke. Neurosci. Lett. 498, 204–207. 10.1016/j.neulet.2011.05.009 21600267

[B8] Bertram J. E. (2005). Constrained optimization in human walking: cost minimization and gait plasticity. J. Exp. Biol. 208, 979–991. 10.1242/jeb.01498 15767300

[B9] Bianco N. A. Patten C. Fregly B. J. (2017). Can measured synergy excitations accurately construct unmeasured muscle excitations? J. Biomech. Eng. 140:011011. 10.1115/1.4038199 29049521

[B10] Bogey R. Hornby T. G. (2007). Gait training strategies utilized in poststroke rehabilitation: are we really making a difference? Top. Stroke Rehabil. 14, 1–8. 10.1310/tsr1406-1 18171655

[B11] Bowden M. G. Balasubramanian C. K. Neptune R. R. Kautz S. A. (2006). Anterior-posterior ground reaction forces as a measure of paretic leg contribution in hemiparetic walking. Stroke 37, 872–876. 10.1161/01.STR.0000204063.75779.8d 16456121

[B12] Cao X. Sui X. Lyu Q. Li L. Chai X. (2015). Effects of different three-dimensional electrodes on epiretinal electrical stimulation by modeling analysis. J. Neuroeng. Rehabil. 12:73. 10.1186/s12984-015-0065-x 26311232 PMC4551567

[B13] Chen G. Patten C. Kothari D. H. Zajac F. E. (2005). Gait differences between individuals with post-stroke hemiparesis and non-disabled controls at matched speeds. Gait Posture 22, 51–56. 10.1016/j.gaitpost.2004.06.009 15996592

[B14] De Groote F. Kinney A. L. Rao A. V. Fregly B. J. (2016). Evaluation of direct collocation optimal control problem formulations for solving the muscle redundancy problem. Ann. Biomed. Eng. 44, 2922–2936. 10.1007/s10439-016-1591-9 27001399 PMC5043004

[B15] De Marchis C. Monteiro T. S. Simon-Martinez C. Conforto S. Gharabaghi A. (2016). Multi-contact functional electrical stimulation for hand opening: electrophysiologically driven identification of the optimal stimulation site. J. Neuroeng. Rehabil. 13:22. 10.1186/s12984-016-0129-6 26955873 PMC4782521

[B16] Delp S. L. Anderson F. C. Arnold A. S. Loan P. Habib A. John C. T.. (2007). OpenSim: open-source software to create and analyze dynamic simulations of movement. IEEE Trans. Biomed. Eng. 54, 1940–1950. 10.1109/TBME.2007.901024 18018689

[B17] Fregly B. J. Boninger M. L. Reinkensmeyer D. J. (2012). Personalized neuromusculoskeletal modeling to improve treatment of mobility impairments: a perspective from European research sites. J. Neuroeng. Rehabil. 9:18. 10.1186/1743-0003-9-18 22463378 PMC3342221

[B18] Garcia-Pinillos F. Cozar-Barba M. Munoz-Jimenez M. Soto-Hermoso V. Latorre-Roman P. (2016). Gait speed in older people: an easy test for detecting cognitive impairment, functional independence, and health state. Psychogeriatrics 16, 165–171. 10.1111/psyg.12133 26114989

[B19] Genthe K. Schenck C. Eicholtz S. Zajac-cox L. Woldf S. Kesar T. M. (2018). Effects of real-time gait biofeedback on paretic propusion and gait biomechanics in individuals post-stroke. Top. Stroke Rehabil. 25, 186–193. 10.1080/10749357.2018.1436384 29457532 PMC5901660

[B20] Hakansson N. A. Kesar T. Reisman D. Binder-Macleod S. Higginson J. S. (2011). Effects of fast functional electrical stimulation gait training on mechanical recovery in poststroke gait. Artif. Organs 35, 217–220. 10.1111/j.1525-1594.2011.01215.x 21401663 PMC3081781

[B21] Hamner S. R. Seth A. Delp S. L. (2010). Muscle contributions to propulsion and support during running. J. Biomech. 43, 2709–2716. 10.1016/j.jbiomech.2010.06.025 20691972 PMC2973845

[B22] He J. Levine W. S. Loeb G. E. (1991). Feedback gains for correcting small perturbations to standing posture. IEEE Trans. Automat. Control 36, 322–332. 10.1109/9.73565

[B23] Jackson J. N. Hass C. J. Fregly B. J. (2016). Development of a subject-specific foot-ground contact model for walking. J. Biomech. Eng. 138:091002. 10.1115/1.4034060 27379886 PMC4967885

[B24] Kesar T. M. Ding J. Wexler A. S. Perumal R. Maladen R. Binder-Macleod S. A. (2008). Predicting muscle forces of individuals with hemiparesis following stroke. J. Neuroeng. Rehabil. 5:7. 10.1186/1743-0003-5-7 18304360 PMC2292738

[B25] Kesar T. M. Reisman D. S. Perumal R. Jancosko A. M. Higginson J. S. Rudolph K. S.. (2011). Combined effects of fast treadmill walking and functional electrical stimulation on post-stroke gait. Gait Posture 33, 309–313. 10.1016/j.gaitpost.2010.11.019 21183351 PMC3042540

[B26] Lamontagne A. Stephenson J. L. Fung J. (2007). Physiological evaluation of gait disturbances post stroke. Clin. Neurophysiol. 118, 717–729. 10.1016/j.clinph.2006.12.013 17307395

[B27] Lee D. D. Seung H. S. (1999). Learning the parts of objects by non-negative matrix factorization. Nature 401, 788–791. 10.1038/44565 10548103

[B28] Liu M. Q. Anderson F. C. Pandy M. G. Delp S. L. (2006). Muscles that support the body also modulate forward progression during walking. J. Biomech. 39, 2623–2630. 10.1016/j.jbiomech.2005.08.017 16216251

[B29] Lloyd-Jones D. Adams R. J. Brown T. M. Carnethon M. Dai S. De Simone G. (2010). Heart disease and stroke statistics-2010 update: a report from the American Heart Association. Circulation 121, e46–e215. 10.1161/CIRCULATIONAHA.109.192667 20019324

[B30] MacKay J. Mensah G. A. (2004). Global Burden of Stroke. Available online at: http://www.who.int/cardiovascular_diseases/en/cvd_atlas_15_burden_stroke.pdf?ua=1 (accessed February 10, 2017).

[B31] McGinley J. L. Morris M. E. Greenwood K. M. Goldie P. A. Olney S. J. (2006). Accuracy of clinical observations of push-off during gait after stroke. Arch. Phys. Med. Rehabil. 87, 779–785. 10.1016/j.apmr.2006.02.022 16731212

[B32] Meyer A. J. Eskinazi I. Jackson J. N. Rao A. V. Patten C. Fregly B. J. (2016). Muscle synergies facilitate computational prediction of subject-specific walking motions. Front. Bioeng. Biotechnol. 4:77. 10.3389/fbioe.2016.00077 27790612 PMC5061852

[B33] Meyer A. J. Patten C. Fregly B. J. (2017). Lower extremity EMG-driven modeling of walking with automated adjustment of musculoskeletal geometry. PLoS ONE 12:e0179698. 10.1371/journal.pone.0179698 28700708 PMC5507406

[B34] Mutikainen S. Rantanen T. Alén M. Kauppinen M. Karjalainen J. Kaprio J.. (2011). Walking ability and all-cause mortality in older women. Int. J. Sport. Med. 32, 216–222. 10.1055/s-0030-1268506 21165808

[B35] Neptune R. R. Kautz S. A. Zajac F. E. (2001). Contributions of the individual ankle plantar flexors to support, forward progression and swing initiation during walking. J. Biomech. 34, 1387–1398. 10.1016/S0021-9290(01)00105-1 11672713

[B36] Neptune R. R. Wright I. C. van den Bogert A. J. (2000). A method for numerical simulation of single limb ground contact events: application to heel-toe running. Comput. Methods Biomech. Biomed. Eng. 3, 321–334. 10.1080/10255840008915275 11264857

[B37] Neptune R. R. Zajac F. E. Kautz S. A. (2004). Muscle force redistributes segmental power for body progression during walking. Gait Posture 19, 194–205. 10.1016/S0966-6362(03)00062-6 15013508

[B38] Nor Azlin N. M. Aziz N. A. A. Saperi B. S. Aljunid S. M. (2016). Functional limitation and health-related quality of life, and associated factors among long term stroke survivors in a Malaysian community. Med. J. Malays. 71, 313–321. 28087954

[B39] Ostir G. V. Berges I. M. Kuo Y. F. Goodwin J. S. Fisher S. R. Guralnik J. M. (2013). Mobility activity and its value as a prognostic indicator of survival in hospitalized older adults. J. Am. Geriatr. Soc. 61, 551–557. 10.1111/jgs.12170 23527951 PMC3628089

[B40] Ostwald S. K. Wasserman J. Davis S. (2006). Medications, comorbidities, and medical complications in stroke survivors: the CAReS study. Rehabil. Nurs. 31, 10–14. 10.1002/j.2048-7940.2006.tb00004.x 16422039 PMC1405842

[B41] Patterson M. A. Rao A. V. (2014). GPOPS– II: a MATLAB software for solving multiple-phase optimal control problems using hp–adaptive Gaussian quadrature collocation methods and sparse nonlinear programming. ACM Trans. Math. Softw. 41, 1–37. 10.1145/2558904

[B42] Perumal R. Wexler A. S. Binder-Macleod S. A. (2008). Development of a mathematical model for predicting electrically elicited quadriceps femoris muscle forces during isovelocity knee joint motion. J. Neuroeng. Rehabil. 5:33. 10.1186/1743-0003-5-33 19077188 PMC2615438

[B43] Pieber K. Herceg M. Paternostro-Sluga T. Schuhfried O. (2015). Optimizing stimulation parameters in functional electrical stimulation of denervated muscles: a cross-sectional study. J. Neuroeng. Rehabil. 12:51. 10.1186/s12984-015-0046-0 26048812 PMC4458019

[B44] Reinbolt J. A. Haftka R. T. Chmielewski T. L. Fregly B. J. (2008). A computational framework to predict post-treatment outcome for gait-related disorders. Med. Eng. Phys. 30, 434–443. 10.1016/j.medengphy.2007.05.005 17616425

[B45] Reinbolt J. A. Schutte J. F. Fregly B. J. Koh B. I. Haftka R. T. George A. D.. (2005). Determination of patient-specific multi-joint kinematic models through two-level optimization. J. Biomech. 38, 621–626. 10.1016/j.jbiomech.2004.03.031 15652563

[B46] Rosso A. L. Verghese J. Metti A. L. Boudreau R. M. Aizenstein H. J. Kritchevsky S.. (2017). Slowing gait and risk for cognitive impairment: the hippocampus as a shared neural substrate. Neurology 89, 336–342. 10.1212/WNL.0000000000004153 28659421 PMC5574674

[B47] Savica R. Wennberg A. M. Hagen C. Edwards K. Roberts R. O. Hollman J. H.. (2017). Comparison of gait parameters for predicting cognitive decline: the Mayo Clinic Study of aging. J. Alzheimers Dis. 55, 559–567. 10.3233/JAD-160697 27662317 PMC5378311

[B48] Schenck C. Kesar T. M. (2017). Effects of unilateral real-time biofeedback on propulsive forces during gait. J. Neuroeng. Rehabil. 14:52. 10.1186/s12984-017-0252-z 28583196 PMC5460355

[B49] Schmid A. Duncan P. W. Studenski S. Lai S. M. Richards L. Perera S.. (2007). Improvements in speed-based gait classifications are meaningful. Stroke 38, 2096–2100. 10.1161/STROKEAHA.106.475921 17510461

[B50] Seth A. Hicks J. L. Uchida T. K. Habib A. Dembia C. L. Dunne J. J.. (2018). OpenSim: simulating musculoskeletal dynamics and neuromuscular control to study human and animal movement. PLoS Comput. Biol. 14:e1006223. 10.1371/journal.pcbi.1006223 30048444 PMC6061994

[B51] Sharma N. Mushahwar V. Stein R. (2014). Dynamic optimization of FES and orthosis-based walking using simple models. IEEE Trans. Neural Syst. Rehabil. Eng. 22, 114–126. 10.1109/TNSRE.2013.2280520 24122568

[B52] Silder A. Whittington B. Heiderscheit B. Thelen D. G. (2007). Identification of passive elastic joint moment-angle relationships in the lower extremity. J. Biomech. 40, 2628–2635. 10.1016/j.jbiomech.2006.12.017 17359981 PMC2020832

[B53] Ting L. H. Chvatal S. A. (2010). Decomposing muscle activity in motor tasks: methods and interpretation, in Motor Control: Theories, Experiments, and Applications, eds Danion F. Latash M. (Oxford: Oxford University Press, 102–138.

[B54] Tresch M. C. Saltiel P. Bizzi E. (1999). The construction of movement by the spinal cord. Nat. Neurosci. 2, 162–167. 10.1038/5721 10195201

[B55] Verma R. Arya K. N. Sharma P. Garg R. K. (2012). Understanding gait control in post-stroke: implications for management. J. Bodyw. Mov. Ther. 16, 14–21. 10.1016/j.jbmt.2010.12.005 22196422

[B56] Wächter A. Biegler L. T. (2006). On the implementation of an interior-point filter line-search algorithm for large-scale nonlinear programming. Math. Program. 106, 25–57. 10.1007/s10107-004-0559-y

[B57] Wenger N. Moraud E. M. Gandar J. Musienko P. Capogrosso M. Baud L.. (2016). Spatiotemporal neuromodulation therapies engaging muscle synergies improve motor control after spinal cord injury. Nat. Med. 22, 138–145. 10.1038/nm.4025 26779815 PMC5061079

[B58] Zarrugh M. Y. Todd F. N. Ralston H. J. (1974). Optimization of energy expenditure during level walking. Eur. J. Appl. Physiol. Occup. Physiol. 33, 293–306. 10.1007/BF00430237 4442409

